# Towards the Application of a Label-Free Approach for Anti-CD47/PD-L1 Bispecific Antibody Discovery

**DOI:** 10.3390/bios13121022

**Published:** 2023-12-09

**Authors:** Artem S. Grevtsev, Alexandra D. Azarian, Alexey K. Misorin, Daria O. Chernyshova, Pavel A. Iakovlev, Mikhail S. Karbyshev

**Affiliations:** JSC “BIOCAD”, 198515 St. Peterburg, Russia; grevtsev@biocad.ru (A.S.G.); misorin@biocad.ru (A.K.M.); yakovlev@biocad.ru (P.A.I.)

**Keywords:** antibodies, bispecific antibodies, label-free, BLI, CD47, PD-L1, kinetic, affinity, reporter, cell-tests

## Abstract

The engineering of bispecific antibodies that exhibit optimal affinity and functional activity presents a significant scientific challenge. To tackle this, investigators employ an assortment of protein assay techniques, such as label-free interaction methodologies, which offer rapidity and convenience for the evaluation of extensive sample sets. These assays yield intricate data pertaining to the affinity towards target antigens and Fc-receptors, instrumental in predicting cellular test outcomes. Nevertheless, the fine-tuning of affinity is of paramount importance to mitigate potential adverse effects while maintaining efficient obstruction of ligand–receptor interactions. In this research, biolayer interferometry (BLI) was utilized to probe the functional characteristics of bispecific antibodies targeting cluster of differentiation 47 (CD47) and programmed death-ligand 1 (PD-L1) antigens, encompassing affinity, concurrent binding to two disparate antigens, and the inhibition of ligand–receptor interactions. The findings derived from BLI were juxtaposed with data from in vitro signal regulatory protein-α (SIRP-α)/CD47 blockade reporter bioassays for two leading bispecific antibody candidates, each demonstrating distinct affinity to CD47.

## 1. Introduction

The past decade has witnessed remarkable advancements in the creation of bispecific antibodies (bsAbs) for therapeutic applications [1]. There are now over 100 distinct bispecific antibody formats at our disposal, each possessing unique characteristics [2] that are predetermined by their potential mechanisms of action and subsequent applications [3,4]. These formats are typically constructed by incorporating the antigen-binding variable fragments (Fv) of two separate antibodies, often in conjunction with multimerization modules.

The intricate process of molecular design and genetic engineering has been leveraged to surmount the technical obstacles associated with the development and production of bispecific antibodies [5]. This encompasses addressing concerns related to functional attributes and the developability profile, taking into account the desired characteristics of the bispecific antibody to be produced, and necessitating the availability of a broad range of formats. These formats can differ in terms of size, configuration, valence, flexibility, and geometry of specific bsAb binding modules, as well as their distribution and pharmacokinetic properties [6].

In certain scenarios, bispecific antibodies are favored over monoclonal antibodies due to their enhanced selectivity and efficacy, as well as their ability to employ complex mechanisms of action that are unattainable by monospecific entities. For instance, immune checkpoint inhibitor antibodies have demonstrated clinical efficacy against a range of malignancies, positioning them as a promising strategy for targeted cancer immunotherapy [7]. However, the presence of resistance in a subset of patients to singular immune checkpoint blockade necessitates the exploration of more complex mechanisms of action. Recent research has indicated that the concurrent inhibition of programmed death protein-1 (PD-1) with programmed cell death ligand-1 (PD-L1) and CD47/SIRP-α immune checkpoint pathways can amplify anti-tumor responses [8].

Meanwhile, the efficacy of the CD47/SIRP-α blockade enacted by monospecific binders is restricted due to inherent toxicity to red blood cells (RBCs) and rapid target-mediated clearance, the latter of which is prompted by the widespread expression of CD47 on normal cells [9]. To surmount these challenges and enhance therapeutic efficacy, CD47/PD-L1 bispecific antibodies have been engineered. Presently, three distinct bispecific antibodies that simultaneously target CD47 and PD-L1 are under development for the treatment of various types of cancer [10,11,12].

The streamlining and fine-tuning bsAbs binding affinities to target proteins provide beneficial opportunities to attenuate unwanted on-target side effects, which is crucial for novel targeted immunotherapeutics [13]. To overcome the mentioned problems directly at the discovery stage, multiple methods for screening protein–protein interactions and functional bioassays are being employed [14]. Several label-free techniques, such as surface plasmon resonance (SPR) [15], biolayer interferometry (BLI) [16], and quartz crystal microbalance (QCM) [17], are instrumental in the selection of suitable lead antibodies for evaluating antibody–antigen interactions. These methodologies can also assess the blocking of ligand–receptor interactions, simultaneous engagement with two targets, epitope binning, and the interaction of the Fc fragment with various receptors. The demand for label-free methods is high during both the early and late stages of biologic drug development due to their rapidity, simplicity, and rich informational yield.

**Scheme 1:**
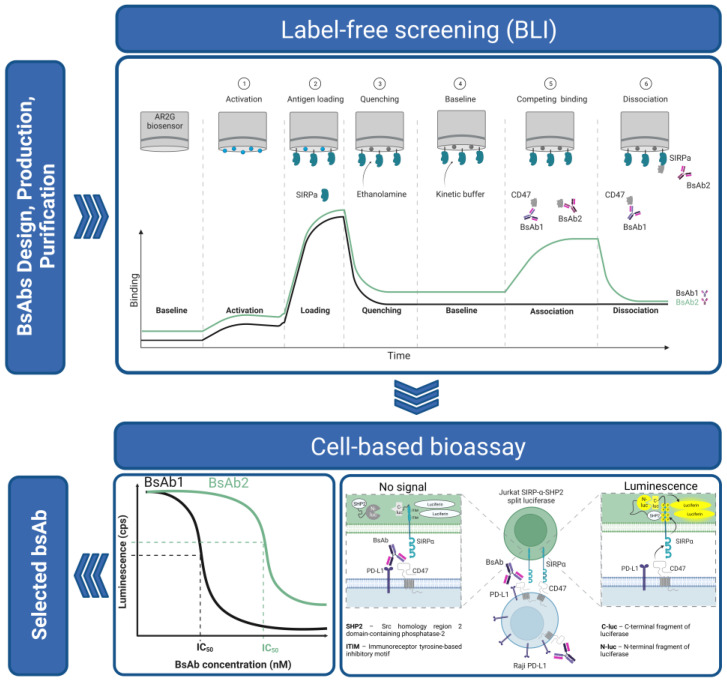
Schematic approach of leading bsAbs selection approach for anti-CD47/PD-L1 bispecific antibody discovery.

This article introduces the use of label-free, biolayer interferometry (BLI)-based methodologies to evaluate critical in vitro attributes of anti-CD47/PD-L1 bispecific antibodies (bsAbs). These methods measure binding affinity, detect bispecific interactions, and evaluate the blocking of CD47-SIRP-α and PD-1/PD-L1 pathways. The results of selecting optimal molecules through the analysis of protein–protein interactions on recombinant proteins may not always align with the findings from cell-based bioassays. This divergence could be attributed to additional physicochemical and biological interaction mechanisms. To ensure the reliability of the results, we have corroborated the findings obtained through the BLI method with primary reporter cell-based tests, which gauge the activation of the SIRP-α signaling pathway (Scheme 1).

## 2. Materials and Methods

### 2.1. Antigen and Antibody Production

All recombinant proteins were expressed in a CHO cell line through transient transfection in-house. Recombinant CD47-FcLama protein contains the CD47 extracellular domain (Leu19-Val134 of DNA sequence NP_942088.1) and Fc fragment of llama IgG. Recombinant proteins PD-L1-hFc-his and PD1-hFc-his have extracellular domains of PDL1 (Phe19-Pro230, NP_054862.1) and PD-1 (Pro21-Val170, NP_005009.2), and both were fused with the C-terminal part of the human IgG Fc and His tag. The recombinant SIRP-α -hFc protein contains the SIRP-α extracellular domain (Glu31-Ser149, NP_542970.1) and C-terminal region of the human IgG Fc. The expressed proteins were purified using protein A MonSelect (BIOCAD, Russia). The eluted proteins were concentrated and then purified using size-exclusion chromatography (HiLoad 16/600 Superdex 75 pg; GE Healthcare, USA). The purified samples were filtered through Millex GP 0.22 µm (Millipore, USA) and stored at −70 °C.

Functional binders to CD47 were procured through phage display using an antibody repertoire derived from a combination of two libraries: one sourced from *Lama glama* immunization and the other from naïve human donors. Specific binding clones were obtained by selecting antigen-binding fragment (Fab) and single-chain variable fragment (scFv) phage libraries on recombinant CD47. Expressed in *Escherichia coli* (*E. coli*) cells, Fab and scFv fragments were screened using an enzyme-linked immunosorbent assay (ELISA) and BLI (Octet RED384, ForteBio, Sartorius AG, Germany). According to the humanization score and affinity to CD47, the best samples were re-cloned in the IgG format and expressed in the CHO. Two samples (binders to CD47) with medium and low affinity (equilibrium dissociation constant (KD) approximately in the range of 10–100 nM and 100–1000 nM, respectively) were chosen for comparative analysis in this work and transferred in a bispecific format.

The PD-L1 binders were obtained using a similar approach. A single high-affinity variant was chosen for comparative analysis. Notably, all the selected binders exhibited a high tolerance for light chain shuffling. To construct the bispecific antibody, a common light chain format and the knob-into-hole Fc heterodimerization approach were utilized. Consequently, the two antibodies investigated in this study, BsAb1 and BsAb2, consisted of an anti-CD47 heavy chain, an anti-PD-L1 heavy chain, and a shared light chain. A monospecific anti-CD47 antibody (Ab3) with a common anti-PD-L1 light chain was obtained as an additional control. Antibodies were produced in Chinese hamster ovary (CHO) cell lines. After clarification via centrifugation, supernatants were purified using protein A MonSelect (BIOCAD, Russia). Neutralized protein A eluates were concentrated and purified by cation exchange chromatography on an SP Sepharose HP (GE Healthcare, USA). After all purification steps, antibody samples were transferred to 20 mM acetate buffer (pH 5.0) with 100 mM trehalose using dialysis cassettes, filtered through Millex GP 0.22 μm (Millipore, USA), transferred to tubes, and stored at −70 °C.

**Figure A1:**
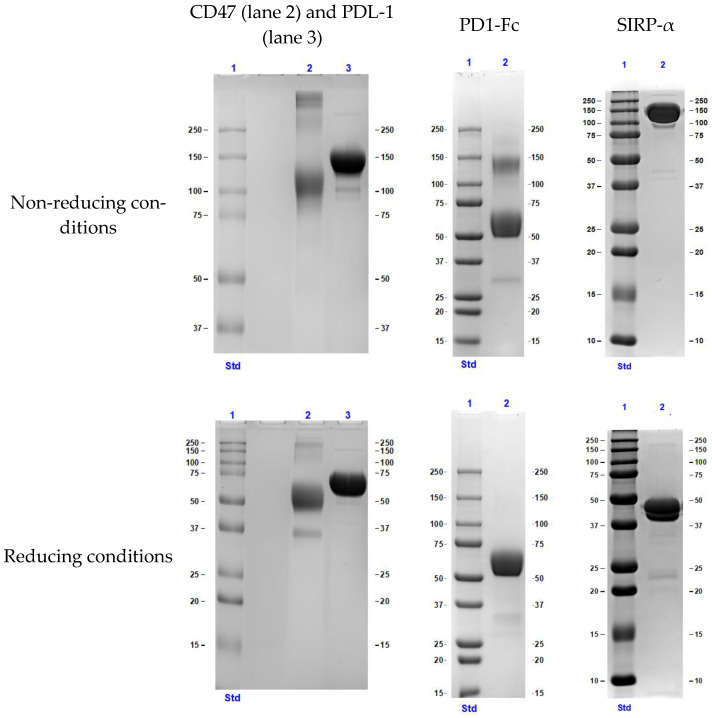
Antigen purity assessment with SDS-PAGE (7.5% polyacrylamide gel under denaturing non-reducing condition and 12.5% polyacrylamide gel under denaturing reducing condition), gels were stained with Coomassie blue R250. Lanes 1—Std, molecular weight marker; Lanes 2 and 3—purified target protein samples.

**Figure A2:**
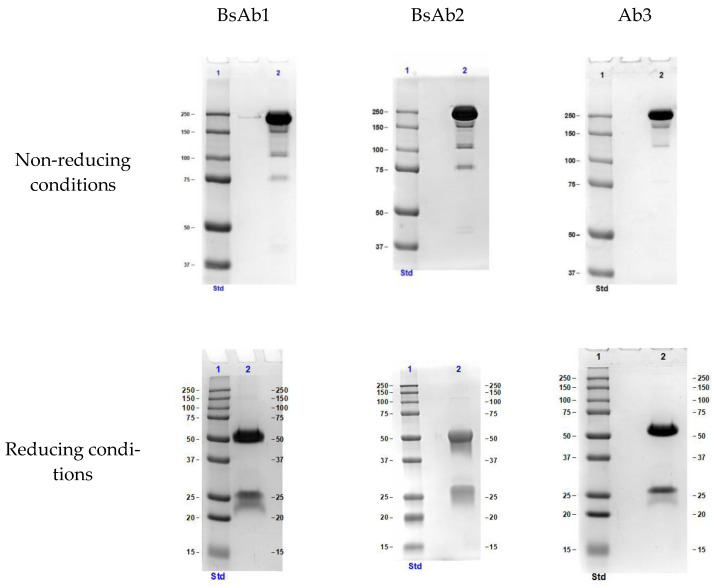
Antibodies purity assessment with SDS-PAGE (7.5% polyacrylamide gel under denaturing non-reducing condition and 12.5% polyacrylamide gel under denaturing reducing condition), gels were stained with Coomassie blue R250. Lanes 1—Std, molecular weight marker; Lanes 2—purified target protein samples.

**Figure A3:**
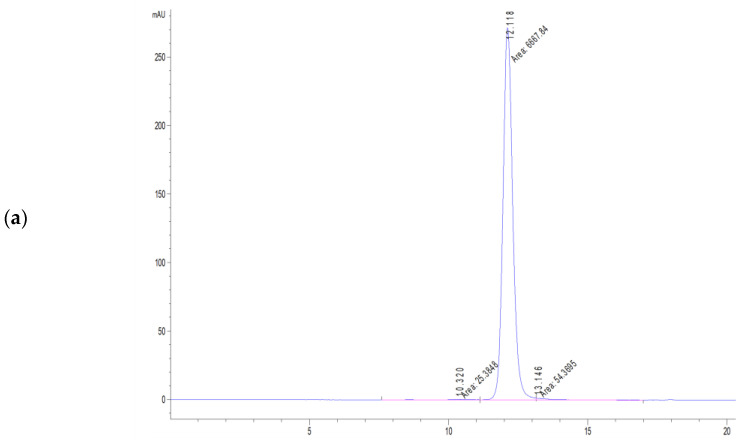

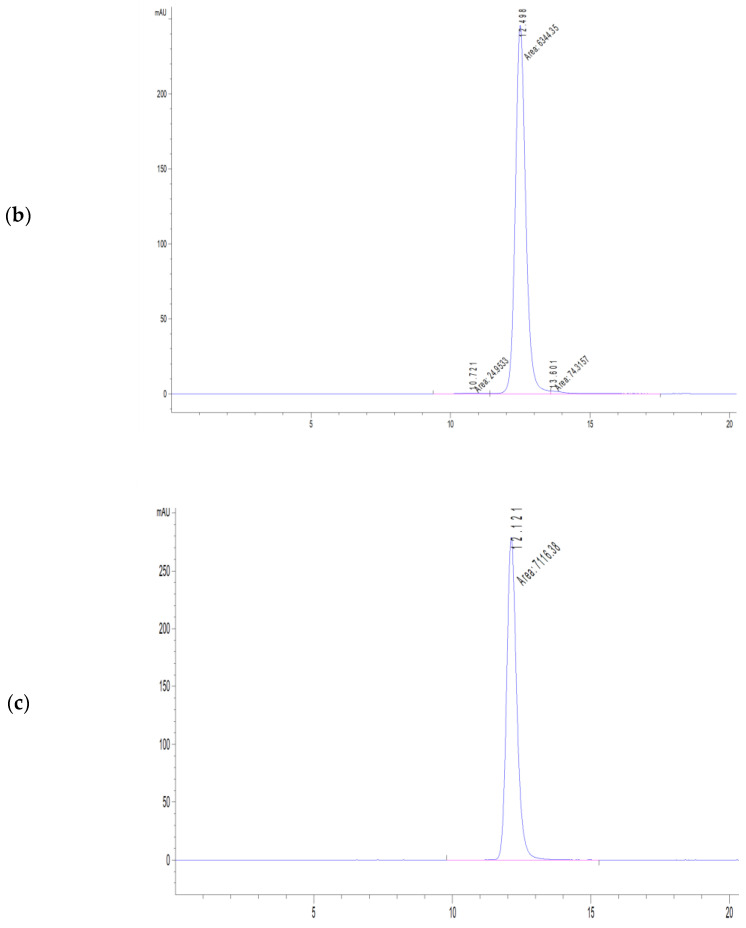
Sample homogeneity assessment with size-exclusion chromatography of BsAb1 (**a**), BsAb2 (**b**), and Ab3 (**c**). Optical density at a wavelength of 280 nm (blue curves) and baseline correction (purple lines) are provided evaluation.

The purity of the samples was assessed with electrophoresis on 7.5% and 12.5% polyacrylamide gels under denaturing non-reducing and reducing conditions (Figure A1 and Figure A2) and size-exclusion high-performance liquid chromatography (HPLC) (Figure A3). Size-exclusion HPLC of the samples was carried out on an Agilent 1100 chromatographic system (Agilent, USA). A TSKgel G3000SWxl 300 mm × 7.8 mm I.D. column (Tosoh, Japan) was used for analysis, and the signal was detected at a wavelength of 280 nm. Isocratic elution mode was used with a flow of 0.7 mL/min in a mobile phase of 100 mM Na_2_HPO_4_ and 200 mM NaCl at pH 6.9.

### 2.2. Antigen Binding Affinity Determination

To determine the equilibrium constant dissociation, association rate constant, and dissociation rate constant of antibodies, a BLI analysis using an OctetRED384 (ForteBio) device was employed. The experiments were performed at 30 °C with orbital agitation at 1000 rpm. The antigen of interest was immobilized on the surfaces of AR2G biosensors (Sartorius AG, Germany). The sensors were activated with a 20 mM *N*-(3-Dimethylaminopropyl)-*N*′-ethylcarbodiimide hydrochloride (EDC) and 10 mM *N*-hydroxysulfosuccinimide (sNHS) mixture in an aqueous solution. After activation, antigens were immobilized in a 10 mM sodium acetate solution at pH 5.0, at a concentration of 10 μg/mL. Unreacted active sites on the sensor surface were quenched for 300 s in a 1 M ethanolamine–HCl solution at pH 8.5. After the quenching stage, all steps were conducted using a kinetic buffer solution consisting of phosphate-buffered saline, 0.1% Tween 20, and 0.1% bovine serum albumin at pH 7.4. A 300 s baseline step was followed by a 60 s association step, during which various concentrations of antibodies were measured using the immobilized antigen. After the association step, the antigen and antibody complex dissociation were detected in a kinetic buffer solution. All measurements were carried out in triplicates.

### 2.3. Binding with Two Antigens CD47 and PD-L1

During the experiment, the sensors were turned on for 300 s in an aqueous solution that contained 20 mM EDC and 10 mM sNHS. To immobilize the PD-L1 antigen on the sensor surface, a solution of 10 mM sodium acetate, pH 5.0, was used for 300 s. The concentration of PD-L1 protein used was 20 μg/mL. The active sites on the sensor surface that were not involved in the reaction were quenched with a 1M ethanolamine–HCl solution at pH 8.5. All the subsequent steps were conducted using a kinetic buffer solution. The antibodies were immobilized onto the sensors with PD-L1 using an antibody concentration of 20 μg/mL for 300 s. During the interaction with CD47, the sensors were immersed in a solution containing 100 μg/mL of CD47. Please refer to Table A1 for a detailed list of the experiment stages. Measurements were carried out in duplicates.

### 2.4. Blocking of PD-L1/PD-1 and CD47/SIRP-α Interaction

To check the blocking of PD-L1/PD-1 interaction, we employed an Octet RED384 instrument from ForteBio on AR2G biosensors made by Sartorius AG. The experiment was conducted at 30 °C with 1000 rpm orbital agitation. Please refer to Table A2 for a detailed list of the experiment stages. The sensors were activated with an aqueous solution containing 20 mM EDC and 10 mM sNHS for 300 s. To load the PD-1 antigen onto the biosensor surface, we used a 10 mM sodium acetate solution (pH 5.0) for 300 s, with a PD-1 protein concentration of 20 μg/mL. To quench any unreacted active sites on the sensor surface, we used a 1 M ethanolamine–HCl solution at pH 8.5 for 300 s. From this point on, all experiment steps were performed in a kinetic buffer solution. At the association stage, the protein-loaded sensors were immersed in wells filled with analyte solutions. The analytes used at this stage were solutions containing 250 nM of the tested antibody and 50 nM PD-L1 in the kinetic buffer. A positive control solution with a PD-L1 concentration of 50 nM was also used. The duration of this stage was 300 s. The experiment to block the CD47/SIRP-α interaction was conducted in a similar way. The SIRP-α was immobilized on sensors. Table A3 presents the steps followed in the experiment. The solution containing 400 nM antibodies and 40 nM CD47 was used as the analyte. A positive control was established using a 40 nM CD47 solution without antibodies. Measurements were carried out in duplicates.

### 2.5. In Vitro SIRP-α/CD47 Blockade Reporter Bioassay

The study aimed to evaluate the ability of test antibodies to block the interaction between the human CD47 and SIRP-α proteins. To achieve this, a bioluminescent cell-based reporter assay was conducted using a genetically modified reporter cell line (Jurkat SIRP-α-Src homology-2-containing protein tyrosine phosphatase 2(SHP2) split luciferase) with a knockout of the CD47 gene (BIOCAD, Russia) and a genetically modified target cell line (Raji) overexpressing human PD-L1 (BIOCAD, Russia).

The Raji (CCL-86) and Jurkat Clone E6-1 (TIB-152) cells were procured from the American Type Culture Collection (ATCC, USA). The PD-L1 overexpressed Raji cell line was generated by transfection using the standard Neon electroporation protocol (Neon Electroporation System, Thermo Fisher Scientific, USA). The CD47 knockout cell line was developed in a Jurkat cell background using Clustered regularly interspaced Short palindromic repeats/CRISPR-associated protein 9 (CRISPR/Cas9) genome editing. Genetic constructs encoding the full-length SIRP-α protein fused to the C-terminal fragment of luciferase, as well as sequentially connected SH1- and SH2-domains of SHP2 phosphatase fused to the N-terminal fragment of luciferase, were inserted into the Jurkat cell line using a standard Neon electroporation protocol (Neon Electroporation System, Thermo Fisher Scientific, USA).

In the experiment, when SIRP-α on the surfaces of Jurkat cells interacts with CD47 on the surfaces of Raji cells, the SHP2 phosphatase within the reporter cell line interacts with the intracellular domain of SIRP-α. This interaction causes the luciferase fragments to combine with these proteins to form a full-length functional enzyme. The introduction of luciferin, the luciferase substrate, into the system leads to the emission of light, which is detected using a plate spectrophotometer. The introduction of anti-CD47 antibody into the system blocks the interaction of CD47 and SIRP-α. As a result, functional luciferase is not assembled, and the luminescence is not detected.

Jurkat SIRP-α-SHP2 split luciferase reporter cells (1 × 10^6^ cells/mL) and Raji PD-L1 target cells (2 × 10^6^ cells/mL) were incubated with serial dilutions of the test antibody at 37 °C for 17 h. The bioluminescent signal was measured using a Bio-Glo Luciferase Assay System (Promega, G7940, USA) and a plate spectrophotometer. The comparison of test antibodies was conducted using equal concentrations of BsAb1 and BsAb2. Independent triplicates were prepared at 9 dilution levels in a range from 6.9 × 10^2^ to 1.0 × 10^−4^ nM for each antibody tested. The point without the test antibody, representing the maximum luminescent signal, was also accessed. The experiment was repeated twice.

### 2.6. Data Analysis

The ForteBio Octet Data Analysis software (version 9.0) was used to process the sensorgrams. For kinetic evaluation, sensorgrams were processed after reference subtraction, applying the 1:1 binding model. The obtained sensorgrams were plotted using Prism 9.5.1 statistical software (GraphPad Software Inc., USA). Affinity values (KD) represented the average of three measurements (±standard deviation). Statistical analysis of the reporter bioassay was performed using Prism 9.5.11 (GraphPad Software Inc., USA). Represented dose–response curves were fitted using the four-parameter logistic model. Each dot represents the mean of three replicates with the standard error of the mean (SEM). Statistical software Prism 9.5.1 (GraphPad Software Inc., USA) was used to determine and plot the half maximum inhibitory concentration (IC50) for BsAb1 and BsAb2 value given the experimental data set.

## 3. Results and Discussion

**Figure 1:**
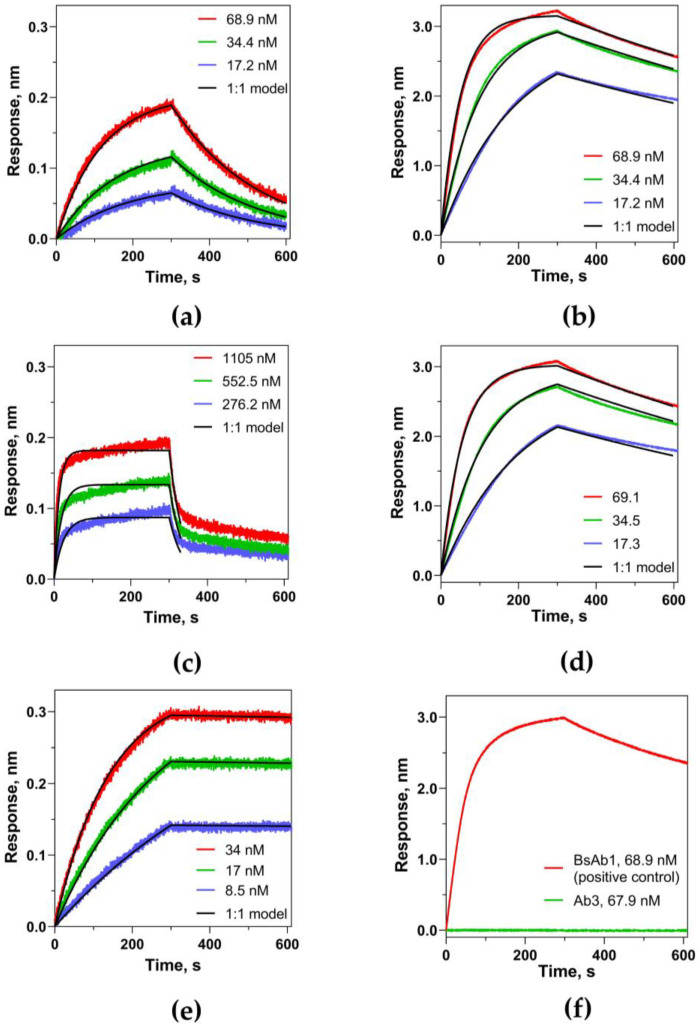
Representative sensorgrams of measurements of antibody affinity to target antigen. Sensorgrams of interaction with CD47 (**a**) BsAb1, (**c**) BsAb2 and (**e**) Ab3 and sensorgrams of interaction with PD-L1 for (**b**) BsAb1, (**d**) BsAb2 and (**f**) Ab3 obtained by BLI on ForteBio Octet384. Measurements were carried out in three replications.

Our interest was aroused by the choice of an antibody that blocks CD47 and SIRP-α binding but at the same time does not have excessive affinity for CD47. We chose candidates with an affinity greater than five times the known affinity of CD47 for SIRP-α, which is approximately 2 μM [18]. As a result, we selected two candidates, BsAb1 and BsAb2, with medium and low affinities, respectively, from the antibodies identified during the early stages of screening and optimization. The results of measuring the kinetics of the interaction of antibodies BsAb1, BsAb2, and the control monospecific antibody to CD47 (Ab3) with CD47 and PD-L1 antigens are presented in Figure 1 and summarized in Table 1.

The affinity of antibody interaction was measured in relation to antigens immobilized on sensors. This measurement method has several advantages for bispecific antibodies. In the case of bispecific antibodies, measurement against an immobilized antigen leads to an interaction corresponding to the 1:1 model for a bivalent (Fc-dimerized) antigen. Meanwhile, there is no nonspecific binding of the analyte (antibodies) to the sensors, even at high concentrations. Based on the kinetics study, BsAb1 exhibited an average affinity of approximately 73.3 ± 8.4 nM, and the observed interaction was well described by a 1:1 binding model. In the case of antibody BsAb2, when fitting the data with a 1:1 model (processing of the dissociation stage was carried out for 30 s due to heterogeneity), the obtained value of the equilibrium dissociation constant KD was 383 ± 44 nM. For BsAb2, interaction with CD47 was observed at higher concentrations compared to BsAb1 due to low affinity. As can be seen in Figure 1c, deviations from the model were observed for this antibody. In the case of a monospecific antibody to CD47, high affinity was observed (KD, when approximated by a 1:1 model, is 0.12 ± 0.05 nM) compared to BsAb1 and BsAb2 due to the bivalent interaction. At the same time, the binding of antibodies BsAb1 and BsAb2 to the PD-L1 antigen was almost identical. The high observed response signal for PD-L1 compared to CD47 may be due to the different number of available binding sites after antigen immobilization. It was well described by the 1:1 interaction model with constants of 2.46 ± 0.10 nM and 2.90 ± 0.08 nM, respectively (Figure 1b,d). The presence of identical affinity for PD-L1 is necessary for the correct comparison of antibodies in a reporter cell test.

**Figure 2:**
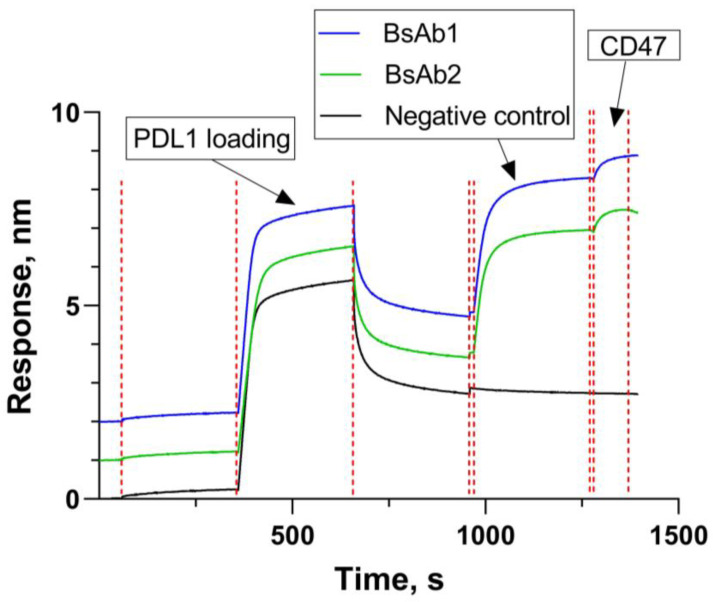
Representative sensorgrams of simultaneous binding with two antigens, CD47 and PD-L1 (the red dotted lines separate the experiment steps; the list of stages with the description is presented in Table A1); measurements were carried out in duplicate.

At the discovery stage of bsAbs development, testing the simultaneous interaction with two different antigens was an actual task to confirm the presence of functional and correctly assembled molecules. The results of the experiment of the simultaneous interaction of antibodies with CD47 and PDL-1 are presented in Figure 2.

During the interaction with CD47, only molecules that have two different binders exhibit a signal. Monospecific antibodies directed against PD-L1 can be loaded onto sensors, but they do not bind to CD47. On the other hand, monospecific antibodies directed against CD47 are not loaded onto sensors during the antibody immobilization step. This experimental setup confirmed the presence of bispecific molecules rather than just a mixture of monospecific antibodies of heavy chains and fragments. One of the significant advantages of this experiment is its capability to identify bispecific molecules even in impure samples. However, interpreting their presence by sodium dodecyl sulfate–polyacrylamide gel electrophoresis (SDS-PAGE) and HPLC information can be challenging without the development of appropriate analytical methods.

This experimental setup can be a convenient tool for analyzing samples from the early stages of screening for bispecific molecules. A similar analysis to test for simultaneous interaction can be carried out using simpler and cheaper methods, such as ELISA, in case of high affinity for both antigens [19]. Studying molecules with low affinity can be quite challenging as they tend to dissociate rapidly. This results in a low level of specific signal being observed after the technical steps of the ELISA analysis, such as interacting with a secondary antibody and washing the plate. Another drawback of ELISA in case of bsAbs is the need for a separate method to detect the second antigen. On the other hand, methods like BLI and SPR that do not require labeling offer a faster assay compared to ELISA.

**Figure 3:**
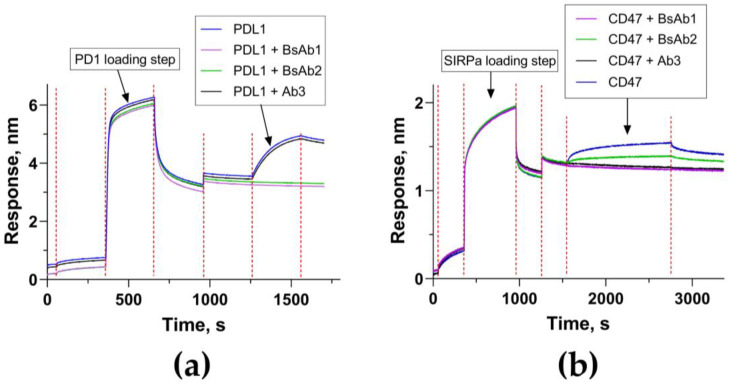
(**a**) Representative sensorgrams of blocking of PD-1/PD-L1 interaction experiments; (**b**) Blocking of CD47/SIRP-α interaction experiment (the red dotted lines separate the stages of the experiment; the list of stages with the description are presented in the Table A2 and Table A3); the measurements were carried out in duplicate.

Testing of the effectiveness of blocking ligand and receptor binding was one of the main ways to compare obtained bispecific antibodies in our case. According to data obtained on OCTET Red384, antibodies BsAb1 and BsAb2 completely blocked the interaction of PD-L1 with PD-1 immobilized on sensors (Figure 3a). The monospecific CD47 antibody Ab3 did not demonstrate any blocking of PD-1/PD-L1 binding, confirming that the anti-CD47 binder of the bsAb does not make a detectable contribution to interaction with PDL-1 antigen. Samples Ab3 and BsAb1 completely blocked CD47/SIRP-α binding, while sample BsAb2 showed only partial blocking of the interaction (Figure 3b) at a molar ratio of anti-CD47 to CD47 epitopes of 5:1.

The results obtained from the kinetics study allowed us to assume that the bispecific antibody BsAb1 can block the activation of the CD47/SIRP-α signal in the intercellular interaction of effector and target cells, while a further decrease in the affinity for CD47 to the level demonstrated by the sample BsAb2 can lead to a significant decrease in the efficiency of blocking the CD47/SIRP-α interaction despite the affinity (345 nM) exceeding the affinity of the interaction of CD47 with SIRP-α (2 µM).

**Figure 4:**
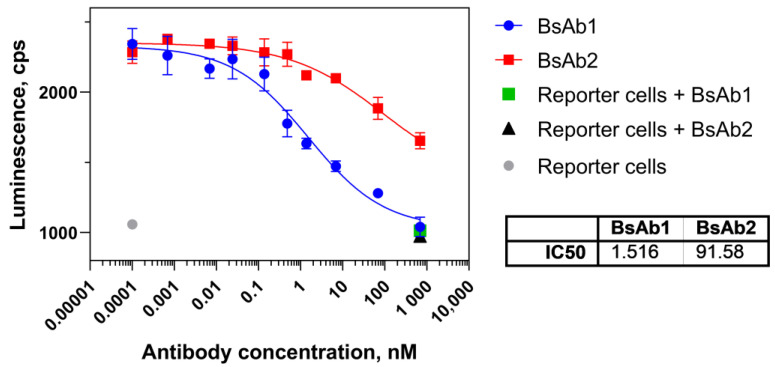
In vitro CD47/SIRP-α interaction axis signaling pathway inhibitory reporter assay; the “Reporter cells + BsAb1”, “Reporter cells + BsAb2”, and “Reporter cells” point signals are comparable to the maximum inhibition point signal; BsAb1 and BsAb2 sample four-parameter models are not parallel.

A model experiment with the target cell line Raji PD-L1 (BIOCAD) and the reporter cell line Jurkat SIRP-α-SHP2 split luciferase CD47KO (BIOCAD) allowed us to compare the effectiveness of antibodies and to test the correlation between the data obtained on recombinant proteins and in cells (Figure 4). Antibody BsAb1 blocks interactions with an IC50 of the order of 1.5 nM; for antibody BsAb2 the estimated IC50 value is higher than 90 nM, while the lower plateau is not reached in the tested range of antibody concentrations. The data obtained are in good agreement with the results of the kinetics study and allow us to confirm the effectiveness of antibody BsAb1 (which has sufficient affinity to block CD47-SIRP-α) and a significant reduction in the blocking effect in antibodies with an affinity of about 350 nM.

## 4. Conclusions

In this study, we conducted a comparative analysis of two prominent anti-CD47/anti-PD-L1 bispecific antibodies during the discovery stage. We employed biolayer interferometry (BLI) to measure the affinity for CD47 and evaluate the blocking of CD47- SIRP-α interaction using recombinant proteins.

Based on the BLI results, it was determined that candidate BsAb1 exhibited a sufficient affinity for CD47, 73.3 ± 8.4 nM, whereas sample BsAb2 displayed incomplete blocking of CD47/SIRP-α and exhibited KD 383 ± 44 nM. The outcomes from the BLI method were consistent with the results obtained from the model reporter cell test, which assessed the blocking of the CD47/SIRP-α signaling pathway. Specifically, BsAb1 demonstrated effective blocking of CD47/SIRP-α with an IC50 of approximately 1.5 nM, while BsAb2 did not reach a lower plateau within the tested range of antibody concentrations (with an IC50 exceeding 90 nM).

Based on these findings, we selected candidate BsAb1 as the appropriate bispecific antibody possessing the necessary affinity for CD47. This selection will serve as a foundation for further developmental stages of the therapeutic bispecific antibody molecule.

## Figures and Tables

**Table 1:** Binding kinetic parameters for antibody–antigen interactions using ForteBio Octet RED384.

Antibody	Target Protein	KD (×10^−9^ M)	k_on_ (×10^5^ M^−1^s^−1^)	k_dis_ (×10^−4^ s^−1^)
BsAb1	CD47	73.3 ± 8.4	0.67 ± 0.08	49.1 ± 4.6
	PD-L1	2.46 ± 0.10	2.77 ± 0.04	6.82 ± 0.17
BsAb2	CD47	383 ± 44	0.63 ± 0.06	239.3 ± 5.4
	PD-L1	2.90 ± 0.08	2.53 ± 0.03	7.34 ± 0.11
Ab3	CD47	0.12 ± 0.05	1.83 ± 0.29	0.22 ± 0.12

The values of equilibrium dissociation constant (KD), association rate constant (k_on_), and dissociation rate constant (k_dis_) represent the average of three independent experiments (±standard deviation).

## Data Availability

Data are contained within the article.

## Appendix A

The purity of the obtained CD47, PDL-1, PD1, and SIRP-α antigens according to electrophoresis under non-reducing conditions was 79.4, 97.3, 65.7, and 91.0%, respectively (79.7, 97.7, 95.4, and 94.2% under reducing conditions). The purity of antibodies BsAb1, BsAb2, and Ab3 according to electrophoresis under non-reducing conditions was 70.6%, 70.1%, and 81.9%, and according to the results of SEC-HPLC it was 98.8, 98.5, and 100%. Samples BsAb1 and BsAb2 had a similar profile of low- and high-molecular-weight impurities and similar purity parameters.

**Table A1:** Stages of binding with two antigens CD47 and PDL-1 binding.

Stage No.	Stage Name	Stage Duration (s)	Comments
1	Baseline 1	60	Sensor check (auxiliary step)
2	Activation	300	For covalent protein immobilization on AR2G sensors
3	Loading PD-L1	300	Covalent immobilization of PD-L1 on the sensor surface
4	Quenching	300	Quenching of unreacted active sites in 1M ethanolamine pH 8.5
5	Baseline 2	10	Sensors washing after quenching step
6	Loading of antibodies	300	Antibodies interact with sensors via a FAB fragment specific to PD-L1
7	Baseline 3	10	Checking of dissociation rate of loaded antibodies (auxiliary step)
8	Association	90	Verification of the interaction of antibodies bound to PD-L1 with the solution of CD47
9	Dissociation	60	To assess the rate of dissociation (auxiliary step)

**Table A2:** Stages of blocking of PD-1–PD-L1 interaction experiment.

Stage No.	Stage Name	Stage Duration (s)	Comments
1	Baseline 1	60	Sensor check (auxiliary step)
2	Activation	300	For covalent protein immobilization on AR2G sensors
3	Loading PD-1	300	Covalent immobilization of PD-1 on the sensor surface
4	Quenching	300	Quenching of unreacted active sites in 1M ethanolamine pH 8.5
5	Baseline 2	300	Equilibration of sensors with kinetic buffer after quenching
6	Association	300	Testing the interaction of the analyte (PD-L1 solution and antibodies) with immobilized PD-1 protein
7	Dissociation	150	To assess the rate of dissociation after interaction with PD-1 (auxiliary step)

**Table A3:** Stages of blocking of CD47–SIRP-α interaction experiment.

Stage No.	Stage Name	Stage Duration (s)	Comments
1	Baseline 1	60	Sensor check (auxiliary step)
2	Activation	300	For covalent protein immobilization on AR2G sensors
3	Loading SIRP-α	600	Covalent immobilization of SIRP-α on the sensor surface
4	Quenching	300	Quenching of unreacted active sites in 1M ethanolamine pH 8.5
5	Baseline 2	600	Equilibration of sensors with kinetic buffer after quenching
6	Association	1200	Testing the interaction of the analyte (solution of CD47 and antibodies) with the immobilized SIRPa antigen
7	Dissociation	600	To assess the rate of dissociation of the positive control (auxiliary step)
